# MAP Kinase Phosphatase-5 Deficiency Protects Against Pressure Overload-Induced Cardiac Fibrosis

**DOI:** 10.3389/fimmu.2021.790511

**Published:** 2021-12-21

**Authors:** Chao Zhong, Kisuk Min, Zhiqiang Zhao, Cheng Zhang, Erhe Gao, Yan Huang, Xinbo Zhang, Margaret Baldini, Rajika Roy, Xiaofeng Yang, Walter J. Koch, Anton M. Bennett, Jun Yu

**Affiliations:** ^1^ Department of Cardiovascular Sciences, Lewis Katz School of Medicine, Temple University, Philadelphia, PA, United States; ^2^ Center for Metabolic Disease Research, Lewis Katz School of Medicine, Temple University, Philadelphia, PA, United States; ^3^ Center for Translational Medicine, School of Traditional Chinese Medicine, Jiangxi University of Chinese Medicine, Nanchang, China; ^4^ Department of Pharmacology, Yale University School of Medicine, New Haven, CT, United States; ^5^ Department of Kinesiology, University of Texas at El Paso, El Paso, TX, United States; ^6^ Department of Internal Medicine, Yale University School of Medicine, New Haven, CT, United States; ^7^ Yale Center for Molecular and Systems Metabolism, Yale University School of Medicine, New Haven, CT, United States

**Keywords:** MKP-5, MAPK signaling, extracellular matrix, macrophages, cardiac fibrosis

## Abstract

Cardiac fibrosis, a pathological condition due to excessive extracellular matrix (ECM) deposition in the myocardium, is associated with nearly all forms of heart disease. The processes and mechanisms that regulate cardiac fibrosis are not fully understood. In response to cardiac injury, macrophages undergo marked phenotypic and functional changes and act as crucial regulators of myocardial fibrotic remodeling. Here we show that the mitogen-activated protein kinase (MAPK) phosphatase-5 (MKP-5) in macrophages is involved in pressure overload-induced cardiac fibrosis. Cardiac pressure overload resulting from transverse aortic constriction (TAC) leads to the upregulation of *Mkp-5* gene expression in the heart. In mice lacking MKP-5, p38 MAPK and JNK were hyperactivated in the heart, and TAC-induced cardiac hypertrophy and myocardial fibrosis were attenuated. MKP-5 deficiency upregulated the expression of the ECM-degrading matrix metalloproteinase-9 (*Mmp-9*) in the Ly6C^low^ (M2-type) cardiac macrophage subset. Consistent with *in vivo* findings, MKP-5 deficiency promoted MMP-9 expression and activity of pro-fibrotic macrophages in response to IL-4 stimulation. Furthermore, using pharmacological inhibitors against p38 MAPK, JNK, and ERK, we demonstrated that MKP-5 suppresses MMP-9 expression through a combined effect of p38 MAPK/JNK/ERK, which subsequently contributes to the inhibition of ECM-degrading activity. Taken together, our study indicates that pressure overload induces MKP-5 expression and facilitates cardiac hypertrophy and fibrosis. MKP-5 deficiency attenuates cardiac fibrosis through MAPK-mediated regulation of MMP*-*9 expression in Ly6C^low^ cardiac macrophages.

## Introduction

Cardiac fibrosis, a common pathophysiologic process associated with most heart diseases, is characterized by excessive extracellular matrix (ECM) deposition and expansion of the interstitium in the myocardium (1, 2). In the fibrotic heart, non-contractile fibrotic tissue ultimately replaces the functional cardiomyocytes and leads to long-term pathological cardiac remodeling, eventually leading to heart failure (3). The ECM proteins that form a cardiac matrix network are integral components for the normal adult mammalian myocardium (4). The collagen-based network of cardiac ECM proteins mainly consists of type I and III collagens, in addition to glycosaminoglycans, glycoproteins, and proteoglycans (1, 2). Importantly, this intricate network of cardiac ECM proteins functions as a scaffold for the myocardium and plays a critical role in transmitting the contractile force (1, 2, 4). In the normal heart, the ECM turnover reaches a homeostatic state between coordinated synthesis and degradation of the fibrillar collagen network (5). However, under pathophysiological conditions, increased collagen deposition in the fibrotic myocardium disrupts the homeostasis of ECM turnover, leading to profound structural and functional impairment of the heart (5). Therefore, targeting cardiac ECM turnover to maintain the balance of collagen homeostasis is very important in preventing and managing cardiac fibrotic remodeling.

Although myofibroblasts are the predominant and direct effector cells (6), macrophages, mast cells, lymphocytes, endothelial cells, and cardiomyocytes also play significant roles in cardiac fibrosis (1, 2). Macrophages are highly heterogeneous, exhibiting strong phenotypic and functional plasticity (7, 8). Depending on the functionally distinct subsets and the microenvironment, macrophages play a wide range of roles in inflammation, tissue repair, and the preservation of immune homeostasis (9–11). Growing evidence has demonstrated that macrophages function as a key regulator of the cardiac fibrotic process (12–15). Two main macrophage populations (Ly6C^high^ and Ly6C^low^) have been implicated in cardiac remodeling, similar to classically activated M1- and alternatively activated M2-type macrophages, respectively (7, 13, 16). During the initial inflammatory phase upon cardiac injury, Ly6C^high^ pro-inflammatory macrophages migrate into the area of the injured heart, along with the secretion of pro-inflammatory cytokines, and function to scavenge and digest necrotic tissue (7, 13, 16). These phagocytic macrophages are important for limiting tissue injury and promoting the reparative transition (16). In the subsequent cardiac healing phase, Ly6C^low^ reparative macrophages contribute to fibrogenesis through mechanisms, including the production of pro-fibrotic cytokines and growth factors, secretion of matrix metalloproteinases (MMPs) and tissue inhibitors, matricellular proteins synthesis, and activation of pro-fibrogenic myofibroblasts (1, 2, 7, 13, 16). Excessive and prolonged activation of reparative macrophages may eventually lead to extensive myocardial fibrosis associated with increased stiffness and cardiac dysfunction (16). However, in addition to the widely recognized pro-fibrotic actions of macrophages, specific macrophage populations may exert anti-fibrotic function. The Ly6C^low^ macrophage population expressing high levels of ECM-degrading MMPs was identified to be responsible for the regression of hepatic fibrosis in mice, and depletion of this macrophage population aggravated fibrosis (17, 18), suggesting the importance of Ly6C^low^ macrophage-derived MMPs in amelioration of tissue fibrotic remodeling. Therefore, it is conceivable that the Ly6C^low^ macrophage population behaves similarly in the remodeling of the myocardium.

MAP kinase phosphatase-5 (MKP-5) belongs to the family of dual-specificity phosphatases (DUSPs) that negatively regulates the activity of the MAP kinases (MAPKs) through direct dephosphorylation of both the regulatory phosphothreonine and phosphotyrosine residues on activated MAPKs (19–21). Evidence has shown that MKP-5 is pivotal in regulating inflammation, immunity, oxidative stress, cancer, insulin resistance, nonalcoholic steatohepatitis, and regenerative myogenesis (19–26). Our previous studies have revealed the involvement of MKP-5 in the pathogenesis of tissue fibrosis (23, 27). Specifically, we have demonstrated that MKP-5 deficiency exerts a protective effect on the progression of muscular dystrophy, including the development of fibrosis in skeletal muscles (23). We also demonstrated that mice lacking MKP-5 are resistant to pulmonary fibrosis in response to bleomycin-induced lung injury (27). Mechanistically, these studies indicated that MKP-5 may positively regulate TGF-β1 signaling, which is an underlying mechanism for pathological fibrogenesis (23, 27). However, the role of MKP-5 in pathological myocardial fibrosis has yet to be determined. Therefore, this study aims to examine the effect of MKP-5 on cardiac fibrotic remodeling and its underlying mechanisms using a mouse model of pressure overload-induced heart failure.

## Materials and Methods

### Mice

Congenic MKP-5-deficient (*Mkp-5^-/-^*) mice were generated and characterized as previously described (21). Briefly, an *Mkp-5* gene targeting vector was transfected into TC-1 mouse embryonic stem cells to replace exon 1 that encodes the first 250 amino acids and its 5’ flanking sequences with a neomycin-resistance gene through homologous recombination. Two homologous recombinants were then injected into C57BL/6 blastocysts. The homozygous *Mkp-5^-/-^* mice were generated using male chimaera mice (21). Wild-type (*Mkp-5^+/+^*) mice were derived from MKP-5 heterozygous mating and propagated subsequently. Eight-week-old male mice were used for all studies. The mice were housed in temperature-controlled rooms with a 12-hour light/12-hour dark cycle and provided free access to food and water throughout the experimental period. All experimental animal procedures were conducted in accordance with guidelines approved by the Institutional Animal Care and Use Committee of Temple University and Yale University.

### Transverse Aortic Constriction (TAC)

TAC surgery was performed as previously described to generate pressure overload in a mouse heart (28). Mice were anesthetized with 3% isoflurane inhalation. The mice were then confirmed to be at a surgical level of anesthesia by the absence of a toe pinch reflex. An incision was made on the center of the chest above the first rib. Muscle layers and thymus tissue are carefully pulled aside to expose the aorta arch. A 7-0 nylon suture was placed around the transverse aorta between the brachiocephalic artery and the left common carotid artery. The aorta is constricted by tightening the suture around a 27-gauge needle, which is then pulled free. The muscle closure was made in one layer with a suture (6-0), followed by skin closure with a non-absorbable polypropylene suture (5-0). Sham-operated control mice were subjected to the identical surgical procedure, including isolation of the aorta, but without suture placement. A pressure gradient over 40 mmHg determined by echocardiography one day after surgery was used as a standard for animals included in the study.

### Myocardial Infarction (MI)

The mouse MI model was performed as previously described (29). Briefly, mice were anesthetized with 2% isoflurane inhalation. A small skin cut (1.2 cm) was made over the left chest and a purse suture was made. After dissection and retraction, a small hole was made at the fourth intercostal space to open the pleural membrane and pericardium. With the clamp slightly open, the heart was smoothly and gently “popped out” through the hole. The left coronary artery was located, sutured and ligated at a site about 3 mm from its origin using a 6-0 silk suture. After ligation, the heart was immediately placed back into the intra-thoracic space followed by manual evacuation of air and closure of muscle and the skin. The mouse was then allowed to breathe room air and monitored during the recovery period.

### Echocardiography

As previously described, transthoracic echocardiography was performed to assess mouse cardiac function at different time points after surgery (13). Mice were anesthetized with 2% isoflurane inhalation and placed on a heated ECG platform. Cardiac function was analyzed by B-mode and M-mode echocardiography using a high-resolution ultrasound system (Vevo3100, Visual Sonics) equipped with an ultrahigh-frequency (38 MHz) linear array transducer. The heart at the left ventricle papillary muscle level was imaged on the left parasternal long and short axis views with a depth setting of 2 cm. Left ventricle posterior wall (LVPW) thickness in diastole/systole, ejection fraction (EF), and fractional shortening (FS) were measured and calculated.

### Histology and Analysis of Cardiac Fibrosis

Mouse hearts were perfused through the left ventricle with ice-cold PBS, followed by perfusion with a 10% KCl solution. The hearts were then perfusion-fixed with 4% paraformaldehyde (PFA, Sigma-Aldrich) for 15 min followed by a further incubation with 4% PFA overnight at 4°C, and subsequently processed for paraffin sections (5 μm in thickness). Heart sections were deparaffinized and hydrated through 100%, 95%, 75%, and 50% ethanol solutions, and then they were used for Sirius Red staining or Masson’s trichrome staining to visualize fibrosis, as previously described (30).

### Fluorescence-Activated Cell Sorting (FACS) Analysis

Mouse hearts were dissected and cut into small pieces following 10 ml ice-cold PBS perfusion. Tissues were then processed for enzymatic digestion with 450 U/ml collagenase I, 125 U/ml collagenase XI, 60 U/ml DNase I, and 60 U/ml hyaluronidase (all Sigma-Aldrich) for 1 h at 37°C with agitation. Subsequently, the heart tissues were triturated and passed through 70 μm cell strainers (BD Falcon), washed, and centrifuged to obtain single-cell suspensions. Next, cells were first enriched for CD11b^+^ cells by using Miltenyi CD11b microbeads and MACS columns according to the manufacturer’s instructions. Then the enriched CD11^+^ cells were stained with antibodies for FACS analysis. The antibodies used were as follows: CD45-APC/Cyanine7 (BioLegend), Ly6G-APC (BioLegend), F4/80-PE (Invitrogen), and Ly6C-BV421 (BioLegend) at 4°C for 30 min. DAPI (ThermoFisher Scientific) was used as a cell viability marker. Flow cytometric analysis and cell sorting were conducted by using a FACSAria™ cell sorter (BD Biosciences). Data were analyzed with FlowJo software.

### Bone Marrow-Derived Macrophage (BMDM) Isolation and Culture

Bone marrow cells were isolated from femurs and tibias of 8-week-old *Mkp-5^+/+^* and *Mkp-5^-/-^* mice as previously described (31). The isolated bone marrow cells were cultured in macrophage growing media (RMPI 1640 supplemented with 10% FBS, 20% L929 fibroblast conditioned media as a source of macrophage colony-stimulating factor, 2 mM L-glutamine, and 1% penicillin/streptomycin) overnight. The next day, non-adherent cells were collected, resuspended, and then cultured with macrophage growing media. Culture media was changed every 2-3 days. Fully differentiated BMDMs were used for studies on day 7.

### Quantitative Real-Time PCR (qRT-PCR)

Total RNA from mouse hearts and cultured cells was isolated using the RNeasy Mini kit (Qiagen) and Trizol reagent (Invitrogen), respectively, according to the manufacturer’s instructions. One microgram of total RNA was used to perform reverse transcription with the iScript™ cDNA synthesis kit (Bio-Rad). qRT-PCR was then conducted using SYBR Green PCR Master Mix (Bio-Rad). cDNA samples were amplified using a CFX96 Touch Real-Time PCR system (Bio-Rad). The relative mRNA levels normalized to *18S* rRNA expression were determined using the comparative ΔΔCt method (32). All the primers used for qRT-PCR are shown in **Supplementary Table 1**.

For fibrotic gene profiling, total RNA from FACS-purified cells from 3 mice in each group was extracted using the RNeasy Micro kit (Qiagen) following the manufacturer’s instructions. A mouse Fibrosis RT^2^ Profiler array (Qiagen) was then used to investigate the expression profile of fibrosis-related genes in FACS-purified Ly6C^low^ cardiac macrophages. First-strand cDNA synthesis, qRT-PCR, and data analysis were performed according to the manufacturer’s protocol as previously described (31).

### Western Blot

Mouse hearts were homogenized and lysed on ice in RIPA lysis buffer supplemented with protease and phosphatase inhibitors (1 mM Na_3_VO_4_, 10 mM NaF, 1 mM benzamidine, 1 mM phenylmethylsulfonyl fluoride, 1 μg/ml pepstatin A, 5 μg/ml aprotinin, 5 μg/ml leupeptin). Cultured cell samples were homogenized in RIPA lysis buffer containing protease and phosphatase inhibitor cocktail (Santa Cruz). The protein concentration was determined using the bicinchoninic acid (BCA) reagent according to the manufacturer’s instructions (Thermo Fisher Scientific). 30 μg of protein was separated by 10-15% SDS-PAGE gel and then transferred to nitrocellulose membranes (Bio-Rad). After blocking with 5% BSA in Tris-buffered saline/Tween-20 (TBST) for 1 h at room temperature, blots were incubated with primary antibodies overnight at 4°C. The following primary antibodies were used: MMP-9 (1:500, Abcam), p-p38 MAPK (1:1000, Cell Signaling), p38 MAPK (1:1000, Cell signaling), p-JNK (1:1000, Cell Signaling), JNK (1:1000, Santa Cruz), p-ERK (1:1000, Cell Signaling), ERK (1:1000, Cell Signaling), HSP90 (1:3000, BD Biosciences). Immunoblots were then incubated with corresponding secondary antibodies (1:5000, LI-COR) for 1 h at room temperature and developed using ODYSSEY Infrared Imaging System (LI-COR). Hsp90 was used as a loading control. Data were quantified with Image Studio software (LI-COR, Version 5.2).

### Gelatin Zymography

Gelatin zymography was performed as previously described (33, 34). Briefly, protein lysates were obtained using ice-cold NP-40 lysis buffer containing protease inhibitors. Equal amounts of protein sample were loaded onto a 10% SDS-PAGE gel supplemented with 0.1% gelatin. After electrophoresis, gels were washed with 0.25% Triton X-100 and incubated with developing buffer (50 mmol/L Tris-HCl, pH 7.4; 5 mM CaCl_2_; 0.02% Brij-35; and 200 mmol/L NaCl) at 37°C for 20 h. Subsequently, the gels were stained using 0.5% Coomassie Brilliant Blue R-250 in 25% isopropanol and 10% glacial acetic acid for 30 min, followed by incubation in 50% methanol and 10% glacial acetic acid for 5-10 min. MMP-digested regions were visualized as unstained, transparent bands against a dark background.

### Statistical Analysis

Data are expressed as mean ± standard errors of the means (SEM). The statistical significance of differences between groups was analyzed using the 2-tailed, unpaired Student’s *t*-test, one-way or two-way analysis of variance (ANOVA) followed by *post-hoc* analysis. A *p*-value < 0.05 was considered as statistically significant. Prism 8 software (GraphPad, CA, USA) was used for graphing and statistical analyses.

## Results

### Pressure Overload Injury Induces *Mkp-5* Expression in the Heart

**Figure 1:**
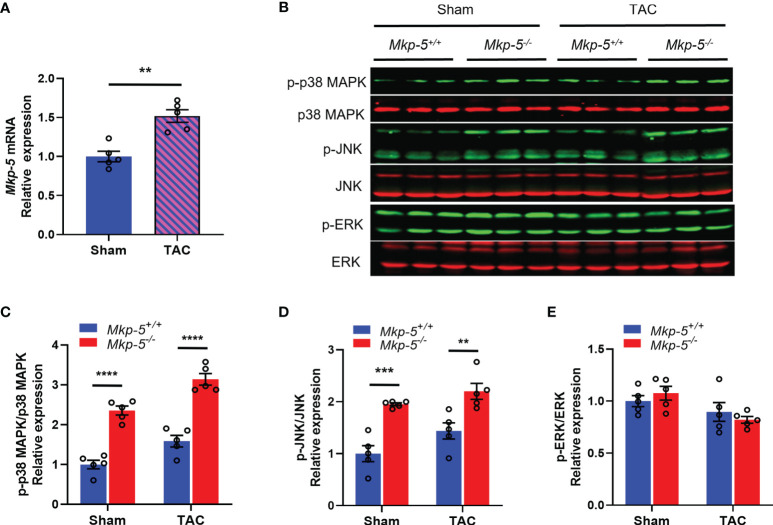
TAC-induced pressure overload increases MKP-5 expression in mouse heart. **(A)** *Mkp-5* mRNA expression levels in mouse hearts were analyzed at 4 weeks after sham or TAC operation (n=5). **P < 0.01 *vs* sham group. **(B)** Western blot analysis of the phosphorylation of p38 MAPK, JNK, and ERK in heart lysates from *Mkp-5^+/+^* and *Mkp-5^-/-^* mice at 4 weeks after sham or TAC operation. **(C–E)** Quantification of phosphorylated p38 MAPK **(C)**, JNK **(D)**, and ERK **(E)** normalized to total p38 MAPK, JNK, and ERK levels in hearts from *Mkp-5^+/+^* and *Mkp-5^-/-^* mice at 4 weeks after sham or TAC operation, respectively (n=5). **P < 0.01, ***P < 0.001, and ****P < 0.0001 *vs Mkp-5^+/+^*. Data are mean ± SEM. Two-tailed, unpaired Student’s *t*-test was used for two group comparison **(A)**. Two-way ANOVA followed by Sidak *post-hoc* test was used for multiple group analysis **(C–E)**.

To examine the potential contribution of MKP-5 in cardiac injury, the expression level of MKP-5 during pathologic remodeling of the heart was examined in mice that underwent sham or TAC surgery. As determined by qRT-PCR analysis, gene expression of *Mkp-5* in the heart (left ventricle) was significantly increased at 4 weeks after TAC-induced pressure overload, compared with those from sham-operated controls (**Figure 1A**). Given that MKPs inactivate the MAPKs through direct dephosphorylation (21), we assessed whether MKP-5 regulates MAPK activity in the mouse left ventricle by measuring the phosphorylation of the MAPKs in cardiac tissues of *Mkp-5^+/+^* and *Mkp-5^-/-^* mice after either sham or TAC surgery. Our data show that the phosphorylation of p38 MAPK and JNK, but not ERK, was significantly increased in the hearts of *Mkp-5^-/-^* mice as compared with *Mkp-5^+/+^* mice in both sham and TAC groups at 4 weeks after surgery (**Figures 1B–E**). These results are consistent with our previous observations showing the effects of MKP-5 on MAPK activity in skeletal muscle (23). Taken together, these data suggest that MKP-5 is activated in the hearts that undergo pressure overload and may be involved in the pathogenesis of heart failure.

### MKP-5 Deficiency Improves Cardiac Function and Attenuates Cardiac Hypertrophy After TAC

**Figure 2:**
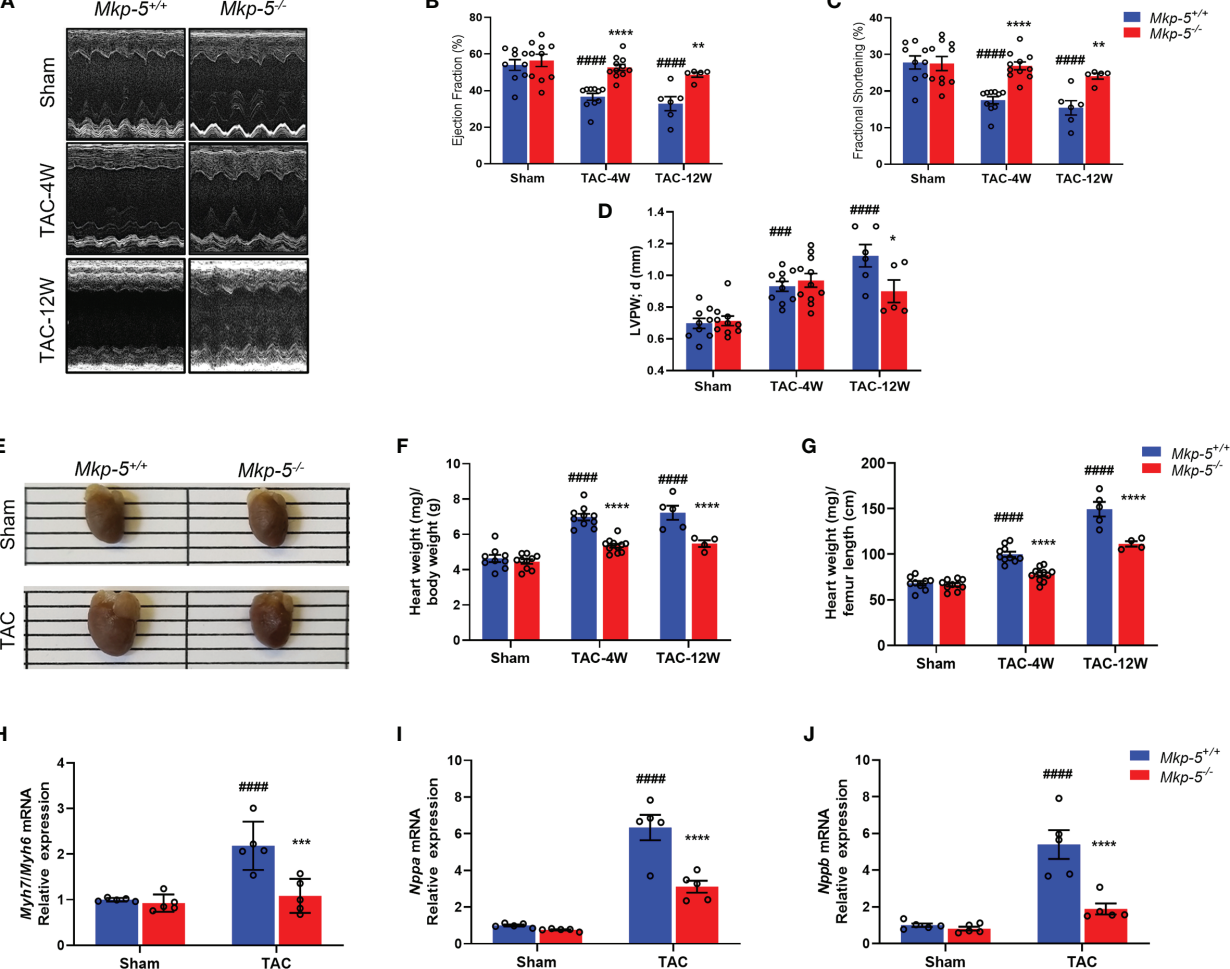
MKP-5 deficiency protects against cardiac dysfunction and cardiac hypertrophy after TAC. **(A)** Representative M-mode tracing for *Mkp-5^+/+^* and *Mkp-5^-/-^* controls at 4 and 12 weeks post TAC or sham operation are shown. **(B–D)** Echocardiographic analysis of ejection fraction **(B)**, fractional shortening **(C)**, left ventricle posterior wall thickness in diastole **(D)** at 4 and 12 weeks after TAC or sham operation (n=5-11). ^###^ *P* < 0.001, and ^####^ *P* < 0.0001 *vs Mkp-5^+/+^* sham, *P < 0.05, **P < 0.01, and ****P < 0.0001 *vs Mkp-5^+/+^* TAC. **(E)** Gross morphology of hearts from *Mkp-5^+/+^* and *Mkp-5^-/-^* mice at 4 weeks after TAC or sham operation. **(F, G)** Heart-to-body weight ratio **(F)** and heart weight-to-femur length ratio **(G)** at 4 weeks and 12 weeks after TAC or sham operation (n=4-11). ^####^ *P* < 0.0001 *vs Mkp-5^+/+^* sham, ****P < 0.0001 *vs Mkp-5^+/+^* TAC. **(H–J)** Gene expression levels of cardiac hypertrophy markers *Myh7*/*Myh6* **(H)**, *Nppa* **(I)**, and *Nppb* **(J)** in hearts from *Mkp-5^+/+^* and *Mkp-5^-/-^* mice at 4 weeks after TAC or sham operation (n=5). ^####^ *P* < 0.0001 *vs Mkp-5^+/+^* sham, ***P < 0.001 and ****P < 0.0001 *vs Mkp-5^+/+^* TAC. Data are mean ± SEM. Two-way ANOVA followed by Sidak *post-hoc* test was used for multiple group analysis **(B–D, F–J)**.

TAC-induced cardiac pressure overload leads to impaired cardiac function and a robust cardiac hypertrophy response in addition to the development of cardiac fibrotic remodeling (35). To evaluate the effects of *Mkp-5* deficiency on cardiac function and cardiac hypertrophy after pressure overload injury, *Mkp-5^+/+^* and *Mkp-5^-/-^* mice were subjected to either TAC or sham surgery. First, we examined cardiac function and remodeling using M-mode echocardiography in sham and TAC mice at 4 and 12 weeks after surgery. As demonstrated by representative echocardiographic images (**Figure 2A**) and analyses (**Figures 2B–D**), MKP-5 deficiency protected against TAC surgery-induced deterioration of left ventricle function as measured by the ejection fraction (EF) and fractional shortening (FS). In addition, left ventricle posterior wall (LVPW) thickening was attenuated by 12 weeks in *Mkp-5^-/-^* mice as compared with those in *Mkp-5^+/+^* pressure overload-induced mice (**Figure 2D**). The echocardiographic parameters in *Mkp-5^-/-^* mice were comparable to those in *Mkp-5^+/+^* controls in sham-operated animals (**Figure 2**) or at baseline level (**Supplementary Table 2**). These results suggest that MKP-5 deficiency protects against cardiac dysfunction induced by pressure overload injury.

Next, cardiac hypertrophy was analyzed in sham- and TAC-operated hearts from *Mkp-5^+/+^* and *Mkp-5^-/-^* mice at 4 and 12 weeks after surgery. As expected, *Mkp-5^+/+^* hearts that underwent 4 weeks of TAC exhibited an increased heart size, indicative of cardiac hypertrophy, as compared with sham-operated controls. However, the heart size of *Mkp-5^-/-^* mice was comparable to sham-operated mice at 4 weeks post TAC (**Figure 2E**). The extent of cardiac hypertrophy was also determined by both the heart-to-body weight ratio and heart weight-to-femur length ratio. At both 4 and 12 weeks after TAC, hearts from *Mkp-5^-/-^* mice showed decreased heart weight-to-body weight ratio as compared with *Mkp-5^+/+^* controls (**Figure 2F**). Similarly, the heart weight-to-femur length ratio was also reduced in *Mkp-5^-/-^* mice (**Figure 2G**). In addition, gene expression levels of cardiac hypertrophy markers *Myh7*/*Myh6*, *Nppa*, and *Nppb* were significantly lower in the hearts from *Mkp-5^-/-^* mice as compared with *Mkp-5^+/+^* controls at 4 weeks after TAC (**Figures 2H–J**). Together, these results demonstrate that MKP-5 deficiency attenuates cardiac hypertrophy after TAC-induced pressure overload injury.

### MKP-5 Deficiency Attenuates Pressure Overload-Induced Cardiac Fibrosis *In Vivo*

**Figure 3:**
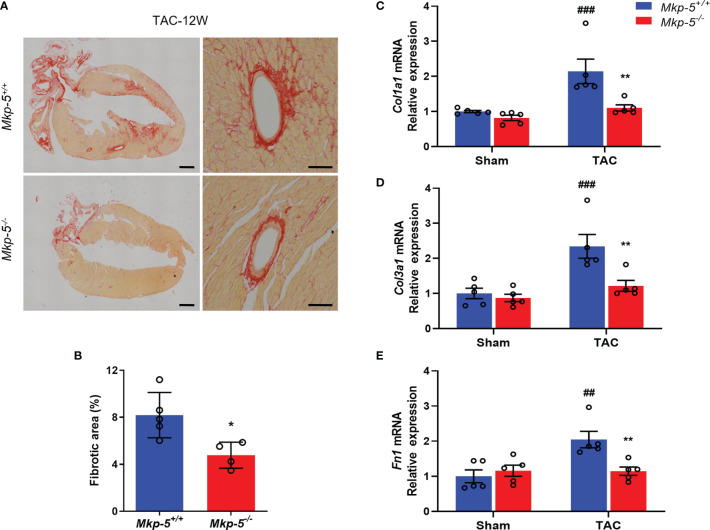
MKP-5 deficiency attenuates cardiac fibrosis after TAC. **(A)** Representative images of Sirius Red-stained sections of *Mkp-5^+/+^* and *Mkp-5^-/-^* mouse hearts at 12 weeks after TAC (scale bar left, 1 mm; scale bar right, 100 μm). **(B)** As indicated by Sirius Red staining, the total cardiac fibrotic area was quantified using sections of *Mkp-5^+/+^* and *Mkp-5^-/-^* mouse hearts at 12 weeks after TAC (n=4-5). *P < 0.05 *vs Mkp-5^+/+^*. **(C–E)** qRT-PCR analysis of *Col1a1*, *Col3a1*, and *Fn1* was performed using mRNA isolated from *Mkp-5^+/+^* and *Mkp-5^-/-^* mouse hearts at 4 weeks after TAC or sham operation (n=5). ^##^ *P* < 0.01 and ^###^ *P* < 0.001 *vs Mkp-5^+/+^* sham, **P < 0.01 *vs Mkp-5^+/+^* TAC. Data are mean ± SEM. Two-tailed, unpaired Student’s *t*-test was used for two group comparison **(B)**. Two-way ANOVA followed by Sidak *post-hoc* test was used for multiple group analysis **(C–E)**.

To investigate the effects of MKP-5 on cardiac fibrotic remodeling, collagen deposition, a hallmark of cardiac fibrosis, was visualized by Sirius Red staining. As expected, TAC-induced pressure overload resulted in significant collagen deposition and myocardial fibrosis as compared with sham-operated *Mkp-5^+/+^* mice (**Supplementary Figure 1**). However, *Mkp-5^-/-^* mice exhibited reduced collagen deposition in cardiac tissues as compared with *Mkp-5^+/+^* controls at 12 weeks post-TAC (**Figures 3A, B**). We further examined the expression of fibrotic ECM genes in the hearts of *Mkp-5^+/+^* and *Mkp-5^-/-^* mice at 4 weeks after TAC. Pressure overload induced a marked increase in the expression of *Col1a1*, *Col3a1*, and *Fn1* in *Mkp-5^+/+^* hearts compared with those from sham-operated mice (**Figures 3C–E**). However, the expression of these fibrogenic genes remained at basal levels in TAC-induced *Mkp-5^-/-^* mice as compared with *Mkp-5^+/+^* controls (**Figures 3C–E**). These findings demonstrate that MKP-5 deficiency attenuates cardiac fibrosis in response to TAC-induced pressure overload. In addition, we confirmed the protective role of MKP-5 deficiency in myocardial fibrosis by using a mouse model of MI. We observed that loss of MKP-5 leads to decreased cardiac fibrosis compared to *Mkp-5^+/+^* controls at 4 weeks after MI (**Supplementary Figure 2**). Thus, MKP-5 deficiency protects against cardiac fibrosis in both pressure overload and an acute ischemic injury model, suggesting that MKP-5 acts as a regulator of myocardial fibrosis.

### MKP-5 Deficiency Promotes the Expression of MMPs in Ly6C^low^ Cardiac Macrophages in Response to Pressure Overload *In Vivo*

**Figure 4:**
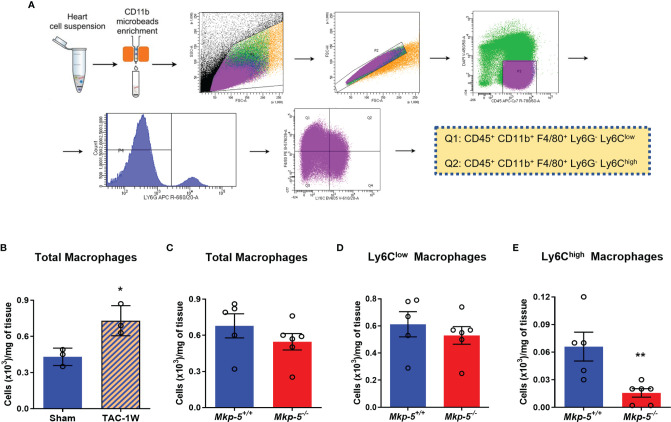
Effects of MKP-5 deficiency on the accumulation of cardiac macrophages in response to TAC. **(A)** Gating strategy for CD45^+^ CD11b^+^ F4/80^+^ Ly6G^-^ Ly6C^low^ and CD45^+^ CD11b^+^ F4/80^+^ Ly6G^-^ Ly6C^high^ cardiac macrophage subsets in hearts after TAC in mice. **(B)** Quantification of total cardiac macrophages from *Mkp-5^+/+^* mice at 1 week after sham or TAC operation (n=3). *P < 0.05 *vs* sham group. **(C–E)** Effects of *Mkp-5* deficiency on total macrophages **(C)**, Ly6C^low^ macrophages **(D)**, and Ly6C^high^ macrophages **(E)** in hearts of mice that underwent 1 week of TAC (n=5-6). Data are mean ± SEM. Two-tailed, unpaired Student’s *t*-test was used for statistical analysis. **P < 0.01 *vs Mkp-5^+/+^*.

Although cardiac fibroblasts and cardiomyocytes contribute to pressure overload induced cardiac fibrosis (1, 2, 6), tissue macrophages also play prominent roles in the regulation of fibrotic remodeling in many different tissues (16, 36, 37) and have been shown to prevent cardiac fibrosis (38). In the current study, we focused on cardiac macrophages and hypothesized that MKP-5 modulates macrophage responses in the heart following pressure overload. Two major macrophage populations Ly6C^high^ and Ly6C^low^ are involved in cardiac remodeling, similar to pro-inflammatory M1- and anti-inflammatory M2-type macrophages, respectively (13). We investigated macrophage responses as a direct consequence of MKP-5 deficiency in mice after TAC. CD45^+^; CD11b^+^; F4/80^+^; Ly6G^-^ Ly6C^+^ macrophages were sorted from hearts derived from *Mkp-5^+/+^* and *Mkp-5^-/-^* mice at 1-week post-TAC (**Figure 4A**), a time point that cardiac macrophage number peaks, as previously described (13, 39). TAC-induced pressure overload increased the number of cardiac macrophages compared with sham-operated controls in *Mkp-5^+/+^* control mice (**Figure 4B**). Loss of MKP-5 showed no significant change in the total number of cardiac macrophages (**Figure 4C**) or Ly6C^low^ (CD45^+^; CD11b^+^; F4/80^+^; Ly6G^-^; Ly6C^low^) cardiac macrophage subset but resulted in a marked decrease in the number of Ly6C^high^ (CD45^+^; CD11b^+^; F4/80^+^; Ly6G^-^; Ly6C^high^) macrophage subset in comparison with the *Mkp-5^+/+^* control mice after TAC (**Figures 4D, E**). Furthermore, MKP-5 deficiency did not influence either Ly6C^low^ or Ly6C^high^ monocyte subsets in the blood of *Mkp-5^+/+^* and *Mkp-5^-/-^* mice in response to TAC (**Supplementary Figure 3**).

**Figure 5:**
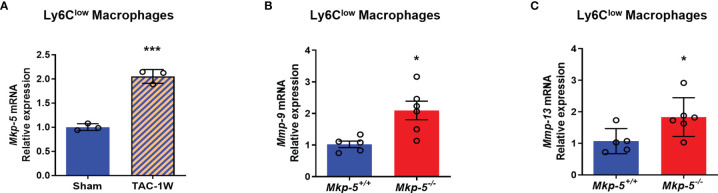
MKP-5 deficiency upregulates the expression of *Mmp-9 and Mmp-13* in Ly6C^low^ cardiac macrophages after TAC. **(A)** Gene expression level of *Mkp-5* in FACS-sorted Ly6C^low^ cardiac macrophages from *Mkp-5^+/+^* mice after 1 week of sham or TAC operation (n=3). ***P < 0.001 *vs* sham group. **(B, C)** qRT-PCR analysis of *Mmp-9* **(B)** and *Mmp-13* **(C)** in Ly6C^low^ cardiac macrophages isolated from a second cohort of *Mkp-5^+/+^* and *Mkp-5^-/-^* mice subjected to 1 week of TAC (n=5-6). *P < 0.05 *vs Mkp-5^+/+^*. Data are mean ± SEM. Two-tailed, unpaired Student’s *t*-test was used for statistical analysis.

Functionally, Ly6C^low^ macrophages are similar to M2-type macrophages, which have been suggested to orchestrate cardiac fibrotic remodeling in response to cardiac injuries such as myocardial infarction and pressure overload (13, 39). To investigate whether MKP-5 is involved in regulating Ly6C^low^ macrophages in response to pressure overload-induced cardiac fibrosis, we examined the expression of MKP-5 in FACS-sorted Ly6C^low^ cardiac macrophages from *Mkp-5^+/+^* and Mkp-5^-/-^ mice after TAC. We found a significant increase in Mkp-5 mRNA expression levels in Ly6C^low^ macrophages isolated from *Mkp-5^+/+^* mice subjected to TAC compared to the sham group (**Figure 5A**). Given that MKP-5 deficiency did not affect the overall number of Ly6C^low^ cardiac macrophages, regulation of fibrosis-related gene expression in Ly6C^low^ macrophages was then examined. We compared the expression profile of fibrosis-related genes in FACS-sorted Ly6C^low^ macrophages from *Mkp-5^+/+^* and *Mkp-5^-/-^* myocardium at 1 week post-TAC using a fibrosis qRT-PCR array. We identified up- and down-regulated fibrosis-modulating genes regulated by MKP-5 in cardiac macrophages (**Supplementary Table 3**). Among these, we found that *Mmp-9* is the most upregulated fibrosis-related gene in Ly6C^low^ cardiac macrophages from *Mkp-5^-/-^* mice after TAC, suggesting the involvement of MMPs in MKP-5-mediated fibrosis. Consistent with this notion, we also observed *Mmp-13* being upregulated in Ly6C^low^ macrophages from *Mkp-5^-/-^* TAC mice. This result was confirmed by qRT-PCR using Ly6C^low^ cardiac macrophages sorted from the second cohort of *Mkp-5^+/+^* and *Mkp-5^-/-^* mice that underwent 1 week of TAC (**Figures 5B, C** and **Supplementary Figure 4**). MMP-9 belongs to the family of MMPs that encode proteolytic enzymes responsible for ECM degradation (17, 18), and importantly, macrophages have been documented as the key source of MMPs (36). Thus, it is conceivable that increased MMP expression in cardiac macrophages could contribute to an enhanced matrix-degrading phenotype and thus, attenuated pressure overload-induced cardiac fibrosis in MKP-5 deficient mice.

### MKP-5 Regulates MMP-9 Expression Through Modulation of MAPK Signaling in Cardiac Macrophage

**Figure 6:**
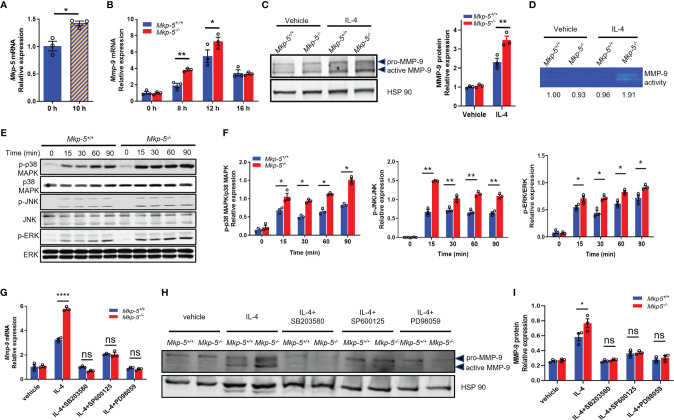
MKP-5 deficiency promotes the expression of MMP-9 in macrophages through MAPK activation. **(A)** Gene expression level of *Mkp-5* in BMDMs stimulated with or without IL-4 (20 ng/ml) for 10 h (n=3 independent isolations). *P < 0.05 *vs* 0 h. **(B)** *Mmp-9* mRNA expression level in *Mkp-5^+/+^* and *Mkp-5^-/-^* BMDMs was analyzed at different time points after stimulation with IL-4 (n=3 independent isolations). *P < 0.05 and **P < 0.01 *vs Mkp-5.* **(C)** MMP-9 protein level in *Mkp-5^+/+^* and *Mkp-5^-/-^* BMDMs was determined by western blotting (Left panel) upon stimulation with or without IL-4 for 24 h. The right panel shows the densitometry quantification (n=3 independent isolations). **P < 0.01 *vs Mkp-5^+/+^*. **(D)** Gelatin zymography analysis of MMP-9 activity using cell lysates from *Mkp-5^+/+^* and *Mkp-5^-/-^* BMDMs treated with or without IL-4 for 24 h Band intensity relative to vehicle-treated macrophages from *Mkp-5^+/+^* is shown. **(E)** Western blot analysis of the phosphorylation of p38 MAPK, JNK, and ERK in *Mkp-5^+/+^* and *Mkp-5^-/-^* BMDMs stimulated with IL-4 for the indicated time periods. **(F)** Quantification of phosphorylated p38 MAPK, JNK, and ERK normalized to total p38 MAPK, JNK, and ERK levels in *Mkp-5^+/+^* and *Mkp-5^-/-^* BMDMs stimulated with IL-4 for indicated time periods (n=3 independent isolations). *P < 0.05 and **P < 0.01 *vs Mkp-5^+/+^*. **(G, H)** qRT-PCR analysis of *Mmp-9* mRNA expression level **(G)** and Western blot analysis of MMP-9 protein level **(H)** in *Mkp-5^+/+^* and *Mkp-5^-/-^* BMDMs stimulated with IL-4 in the presence or absence of p38 MAPK-, JNK-, or ERK-specific inhibitors (SB203580, SP600125, or PD98059) (n=3 independent isolations). ****P < 0.0001 *vs Mkp-5^+/+^*. **(I)** Quantification of MMP-9 protein expression normalized to Hsp90 level in *Mkp-5^+/+^* and *Mkp-5^-/-^* BMDMs stimulated with IL-4 in the presence or absence of p38 MAPK-, JNK-, or ERK-specific inhibitors (SB203580, SP600125, or PD98059). (n=3 independent isolations). *P < 0.05 *vs Mkp-5^+/+^*. Data are mean ± SEM. Two-tailed, unpaired Student’s *t*-test was used for two group statistical comparison **(A)**. Two-way ANOVA followed by Sidak *post-hoc* test was used for multiple group analysis **(B, C, F, G, I)**. “ns” indicates no statistical difference between two groups.

To investigate the mechanisms by which MKP-5 regulates the expression of MMP-9 in macrophages, IL-4-stimulated BMDMs were used as a pro-fibrotic polarization model (13). Treatment with IL-4 for 10 h significantly induced the mRNA expression of *Mkp-5* in BMDMs (**Figure 6A**). Consistent with the *in vivo* findings that TAC upregulates *Mkp-5* in the heart (**Figure 1A**) and Ly6C^low^ cardiac macrophages (**Figure 5A**), these results suggest that MKP-5 is responsive to pro-fibrotic stimuli. IL-4 stimulation increased *Mmp-9* mRNA level in a time-dependent manner with a peak expression at 12 h in BMDMs (**Figure 6B**). Consistent with our qRT-PCR array data using FACS-sorted Ly6C^low^ cardiac macrophages, significant upregulation of MMP-9 expression at both mRNA and protein levels was observed in *Mkp-5^-/-^* BMDMs as compared with *Mkp-5^+/+^* controls after IL-4 stimulation (**Figures 6B, C**). In agreement with these observations, the gelatin zymography assay showed a marked increase of MMP-9 enzymatic ECM-degrading activity in cell lysates derived from *Mkp-5^-/-^* BMDMs compared with *Mkp-5^+/+^* controls in response to IL-4 (**Figure 6D**).

MKP-5 has been shown to dephosphorylate p38 MAPK, JNK, and ERK to a lesser extent (40, 41). The effects of MKP-5 deficiency on the activation of the MAPKs were examined in IL-4-treated BMDMs. In response to IL-4 stimulation, *Mkp-5^-/-^* BMDMs exhibited enhanced phosphorylation of p38 MAPK, JNK, and to a lesser extent ERK as compared with *Mkp-5^+/+^* controls (**Figures 6E, F**). These results demonstrate that MKP-5 negatively regulates p38 MAPK and JNK activities in IL-4-stimulated M2-type macrophages and results in upregulation of *Mmp-9* expression. Previous studies have shown that the activated MAPKs (p38 MAPK, JNK, and ERK) can promote MMP-9 expression in macrophages (42). We therefore examined whether MKP-5 regulates MMP-9 in a MAPK-dependent manner in BMDM. After treatment with p38 MAPK-, JNK-, or ERK-specific inhibitors SB203580, SP600125, or PD98059, respectively, the increase of MMP-9 expression induced by IL-4 in *Mkp-5^-/-^* BMDMs relative to *Mkp-5^+/+^* controls was blocked at both mRNA and protein levels (**Figures 6G–I**). Collectively, these data indicate that MAPK signaling is required for MKP-5 mediated regulation of MMP-9 in pro-fibrotic macrophages. Together with the *in vivo* MKP-5 loss of function studies, these data reveal a regulatory pathway by which MKP-5 inhibits the expression of MMP-9 by inactivating MAPK signaling in Ly6C^low^ M2-type macrophages, which subsequently leads to an attenuated ECM-degrading activity and thus, at least in part, development of cardiac fibrosis induced by pressure overload.

## Discussion

Myocardial fibrosis is characterized by ECM production and degradation imbalance, contributing to cardiac dysfunction and further deterioration of heart failure. Although significant advances have been made towards understanding the cellular and molecular mechanisms underlying the pathogenesis of cardiac fibrosis, clinical translation of this knowledge to treat heart disease has yet to be fully realized. Macrophages have been viewed as one of the central players of cardiac fibrosis, strengthening the macrophage-based hypothesis of cardiac fibrosis (38, 43). In this study, we identified MKP-5, along with its regulated Ly6C^low^ macrophage-derived ECM-degrading MMP-9, as coordinators of cardiac fibrosis in response to TAC-induced hypertensive injury. We have determined that MKP-5 deficiency attenuates cardiac fibrotic remodeling, preserves cardiac function, and prevents cardiac hypertrophy in mice subjected to TAC-induced pressure overload injury. Therefore, modulation of this pathway may provide a novel strategy towards preventing pressure overload-induced cardiomyopathy.

In response to pressure overload, our data demonstrated that *Mkp-5* gene expression is significantly upregulated in the myocardium (**Figure 1**), suggesting a regulatory role of MKP-5 in pressure overload-induced cardiac remodeling. Notably, our previous studies have demonstrated that MKP-5 is upregulated in response to damage and promotes fibrosis in skeletal muscle and lung (23, 27). Here, using loss-of-function approaches, we show that MKP-5 deficiency exerts a protective effect on cardiac fibrosis by inhibiting excessive collagen deposition in both pressure overload and acute ischemic injury models (**Figure 3** and **Supplementary Figure 2**), demonstrating a requirement for MKP-5 in regulating cardiac fibrosis in response to cardiac injury. Thus, these findings further extend the role of MKP-5 in the regulation of tissue fibrosis. In addition, we observed that MKP-5 deficiency attenuated cardiac hypertrophy and improved cardiac function after TAC (**Figure 2**). It has been previously reported that inhibition of cardiac fibrosis could lead to reduced cardiomyocyte hypertrophy and improved cardiac function (34, 44). Furthermore, it has been documented that myocardial fibrosis may act as an early manifestation of hypertrophic cardiomyopathy in mice or human patients with pathogenic sarcomere protein mutations that cause familial hypertrophic cardiomyopathy (45, 46). These studies provide evidence for the potential role of fibrosis in the progression of pressure overload-induced cardiac dysfunction and failure. Therefore, while we cannot fully rule out the role of MKP-5 in cardiomyocytes in mediating heart failure, the reduced cardiac fibrosis may potentially contribute to the preserved heart failure phenotype in our current study.

Macrophages are highly plastic and functionally diverse depending upon the physiological status and following pathological insults (7, 8). Abundant evidence has demonstrated that macrophages are critically involved in cardiac fibrotic remodeling (16, 37, 38). Upon cardiac injury, Ly6C^high^ M1-like macrophages accumulate in injured sites, release high levels of pro-inflammatory mediators, scavenge and digest dead tissue or necrotic debris (7, 13, 31). In contrast, the alternatively-activated Ly6C^low^ M2-like macrophages promote repair and induce fibrosis through mechanisms including the production of pro-fibrotic cytokines and growth factors, secretion of MMPs and their tissue inhibitors to modify ECM turnover, and activation of cardiac fibroblasts to deposit scar-forming collagen (7, 13, 31). In this study, we showed that *Mkp-5* expression is markedly increased in Ly6C^low^ cardiac macrophages in response to pressure overload, and MKP-5 deficiency leads to a significant increase in *Mmp-9* in Ly6C^low^ cardiac macrophages (**Figures 4**, **5**). Ly6C^low^ cardiac macrophages are rich sources of MMPs (36), and MMP-9 facilitates ECM degradation (17, 18). It is thus reasoned that the reduced cardiac fibrosis in MKP-5 deficient mice is attributed, at least in part, to the increased expression of *Mmp-9* in Ly6C^low^ cardiac macrophages. Indeed, our results are supported by previous studies using various experimental animal models of cardiac remodeling. Increased expression of MMP-9 was found to be associated with attenuated cardiac fibrosis and preserved cardiac function (44–50). Importantly, the elevated MMP-9-mediated collagen degradation resulting from the increased cardiac MMP-9 expression was able to inhibit adverse fibrotic remodeling of the myocardium (44–50). In addition, MMP-9 also exerts cardioprotective effects independent of its direct ECM-degrading function. Macrophage-specific overexpression of MMP-9 improved left ventricle function and decreased ECM synthesis by attenuating the inflammatory response after myocardial infarction (51). Our results showed that MKP-5 deficiency decreased Ly6C^high^ pro-inflammatory cardiac macrophage accumulation (**Figure 4**) and increased MMP-9 expression in Ly6C^low^ macrophages after TAC (**Figure 5** and **Supplementary Table 3**). It is thus reasonable to propose that MKP-5 deficiency protects against pressure overload-induced cardiac remodeling, at least in part, through a mechanism involving macrophage MMP-9-mediated attenuation of the cardiac inflammatory response. However, other studies of MMP-9 in cardiac remodeling have come to different conclusions. For example, the MMP-9 global knock-out mouse, when subjected to experimental models of myocardial infarction, exhibited increased survival with improved cardiac repair and reduced fibrotic remodeling (52, 53), which is similar to the phenotype found in macrophage MMP-9 overexpressing transgenic mice (51). The reconciliation of these data may be that MMP-9 plays different roles depending on the cellular source and the physiological or pathological context (54). These observations also imply that the entirety of the MKP-5-mediated effects on cardiac fibrosis is not simply mediated through MMP-9 alone, and likely other factors play contributing roles.

Although our data suggests that MKP-5 in macrophages promotes pressure overload-induced cardiac fibrosis, future studies using bone marrow transplantation or macrophage-specific MKP-5 knock-out mice will be necessary to further substantiate these results. We cannot exclude the possibility that MKP-5 in other cardiac cell types also contributes to the development of the phenotypes observed in MKP-5-deficient mice. Indeed, our previous studies have demonstrated that MKP-5 in lung fibroblasts plays a crucial role in developing pulmonary fibrosis (27). A previous study has also shown that cardiomyocyte overexpression of MKP-1, another DUSP family member, attenuated hypertrophy in response to pressure overload (55). Thus, the role of MKP-5 in cardiac fibroblasts and cardiomyocytes in the development of myocardial fibrosis and heart failure is worthy of further investigation. Furthermore, the interaction between macrophages and cardiac fibroblasts during cardiac remodeling has gained growing attention (12). Macrophage-fibroblast crosstalk constitutes a regulatory loop by which macrophages secrete pro- and/or anti-fibrotic mediators that in turn stimulate and/or attenuate cardiac fibroblast activation and ECM deposition. Whether this interaction is involved in MKP-5-mediated cardiac fibrotic remodeling has yet to be determined.

MKP-5 is a dual-specificity phosphatase that directly dephosphorylates and inactivates p38 MAPK and JNK but with a lower preference for ERK (40, 41). Consistent with previous observations, we showed that MKP-5 deficiency leads to enhanced phosphorylation of p38 MAPK and JNK in heart lysates (**Figures 1**, **6**). Interestingly, we observed increased phosphorylation of ERK in *Mkp-5^-/-^* BMDMs upon stimulation by IL-4, although to a lesser extent as compared with either p38 MAPK or JNK, indicating that ERK is also a target of *Mkp-5* under these conditions. Our results are in accordance with a previous report that shows MKP-5 inactivates p38 MAPK, JNK, and ERK in obesity-related inflammation (56). Therefore, these findings may reflect the fact that MKP-5 has context-dependent substrate selectivity (57). In addition, genetic deletion or pharmacological inhibition of p38 MAPK, JNK, and ERK has been shown to promote hypertrophic or ischemic cardiomyopathy, indicating the cardioprotective effects of the MAPKs (58–61). Our data showed that MKP-5 deficiency results in hyperactivation of the MAPKs and exerts a beneficial effect on hypertensive heart failure, which provides further insight into the potential mechanism of MKP-5-mediated effects in cardiac remodeling during pressure overload. Moreover, our current study provides evidence demonstrating that MKP-5-dependent regulation of MMP-9 is mediated by p38 MAPK and, to a lesser extent, by JNK and ERK. Consistent with our observation, the expression of MMP-9 has been previously reported to be induced *via* activation by the MAPKs (p38 MAPK, JNK, and ERK), and pharmacological inhibition or gene silencing of the MAPKs suppressed MMP-9 expression (42, 62–64). Thus, our results implicate MKP-5 as part of the *Mmp-9* axis that regulates ECM turnover which contributes to cardiac fibrosis.

In summary, we demonstrate that activation of MKP-5 is required to promote pressure overload-induced cardiac fibrosis. We propose a model in which MKP-5 dephosphorylates p38 MAPK, JNK, and ERK, thereby suppressing the expression of ECM-degrading MMP-9 in Ly6C^low^ M2-like macrophages in the heart following pressure overload-induced injury. This study extends our understanding of MKP-5 as a regulator of fibrotic signaling and reveals its involvement in myocardial remodeling.

## Data Availability Statement

The original contributions presented in the study are included in the article/**Supplementary Material**. Further inquiries can be directed to the corresponding authors.

## Ethics Statement

The animal study was reviewed and approved by IACUC committee of Temple University and Yale University.

## Author Contributions

AB and JY designed the study and coordinated all experimental work. CZho, KM, ZZ, CZha, EG, YH, XZ, MB, and RR carried out the experimental work. CZho, KM, ZZ, XY, WK, AB, and JY analyzed and interpreted the data. CZho, KM, AB, and JY wrote the manuscript with valuable input from all other authors. All authors contributed to the article and approved the submitted version.

## Funding

This work was supported by grants from the National Institutes of Health (NIH) R01 HL126933 and HL153599, and an Award from American Heart Association (18EIA33900065) to JY. AB was supported by R01 HL134166. CZho was supported by grants from the Research Start-up Fund of Jiangxi University of Chinese Medicine (2018BSZR002) and the First-Class Discipline Development Program (JXSYLXK-ZHYI054).

## Conflict of Interest

The authors declare that the research was conducted in the absence of any commercial or financial relationships that could be construed as a potential conflict of interest.

## Publisher’s Note

All claims expressed in this article are solely those of the authors and do not necessarily represent those of their affiliated organizations, or those of the publisher, the editors and the reviewers. Any product that may be evaluated in this article, or claim that may be made by its manufacturer, is not guaranteed or endorsed by the publisher.
