# Supplementary material for: MAP Kinase Phosphatase-5 Deficiency Protects Against Pressure Overload-Induced Cardiac Fibrosis
Source: Front Immunol. 2021 Dec 21;12:790511. doi: 10.3389/fimmu.2021.790511 (PMC8724134; doi:10.3389/fimmu.2021.790511)
Supplement: Supplementary file 1 [file DataSheet_1.pdf]

Chao Zhong[1,2,3,#], Kisuk Min[4,5,#], Zhiqiang Zhao[1,2], Cheng Zhang[1,2], Erhe Gao[1], Yan Huang[6], Xinbo Zhang[6], Margaret Baldini[1,2], Rajika Roy[1], Xiaofeng Yang[1], Walter J Koch[1], Anton M. Bennett[4,7,*], and Jun Yu[1,2,*]

[1]Department of Cardiovascular Sciences, Lewis Katz School of Medicine, Temple University, Philadelphia, PA, USA.
[2]Center for Metabolic Disease Research, Lewis Katz School of Medicine, Temple University, Philadelphia, PA, USA.
[3]Center for Translational Medicine, School of Traditional Chinese Medicine, Jiangxi University of Chinese Medicine, Nanchang, Jiangxi, China.
[4]Department of Pharmacology, Yale University School of Medicine, New Haven, CT, USA
[5]Department of Kinesiology, University of Texas at El Paso, El Paso, TX, USA
[6]Department of Internal Medicine, Yale University School of Medicine, New Haven, CT, USA
[7]Yale Center for Molecular and Systems Metabolism, Yale University School of Medicine, New Haven, CT, USA

[#]These authors contributed equally to this work.

***Correspondence:**

Jun Yu, MD
Department of Cardiovascular Sciences, Center for Metabolic Disease Research,
Lewis Katz School of Medicine, Temple University, Philadelphia, PA 19140, USA.
Email: jun.yu@temple.edu

Anton M. Bennett, PhD
Department of Pharmacology, Yale University School of Medicine, New Haven, CT
Email: anton.bennett@yale.edu

Supplementary Table 1: Sequences of qRT-PCR primers used in this study

Gene	Forward (5’-> 3’)	Reverse (5’-> 3’)
Mkp-5	GCAACCTACGACAAGGATCATC	GTTGACCATCTGGTTAGCAGG
Myh6	GCCCAGTACCTCCGAAAGTC	GCCTTAACATACTCCTCCTTGTC
Myh7	GAGCAAGGCCGAGGAGACGCAGCGT	GAGCCTCCTTCTCGTCCAGCTGCCGG
Nppa	CCTGGAGGAGAAGATGCCGGTAGAA	CCCCAGTCCAGGGAGGCACCTCGG
Nppb	CACTTCAAAGGTGGTCCCAGAGCTGC	GACCGGATCGGATCCGTCAGTCG
Col1a1	TCCTGACGCATGGCCAAGAAGACA	TCCGGGCAGAAAGCACAGCACTC
Col1a2	GGTGAGCCTGGTCAAACGG	ACTGTGTCCTTTCACGCCTTT
Col3a1	GCACAGCAGTCCACCGTAGA	TCTCCAAATGGGATCTCTGG
Fn1	ATGTGGACCCCTCCTGATAGT	GCCCAGTGATTTCAGCAAAGG
MMP-9	GGACCCGAAGCGGACATTG	CGTCGTCGAAATGGGCATCT
MMP-13	CTTCTTCTTGTTGAGCTGGACTC	CTGTGGAGGTCACTGTAGACT
Plau	GCGCCTTGGTGGTGAAAAAC	TTGTAGGACACGCATACACCT
Dcn	TCTTGGGCTGGACCATTTGAA	CATCGGTAGGGGCACATAGA
Vegfa	GCACATAGAGAGAATGAGCTTCC	CTCCGCTCTGAACAAGGCT
Thbs1	GGGGAGATAACGGTGTGTTTG	CGGGGATCAGGTTGGCATT
Stat1	TCACAGTGGTTCGAGCTTCAG	GCAAACGAGACATCATAGGCA
Itgb3	CCACACGAGGCGTGAACTC	CTTCAGGTTACATCGGGGTGA
18S rRNA	TTCCGATAACGAACGAGACTCT	TGGCTGAACGCCACTTGTC

Supplementary Table 2: Baseline cardiac function of *Mkp-5*$^{+/+}$ and *Mkp-5*$^{-/-}$ mice measured by echocardiography

Parameter	***Mkp-5*$^{+/+}$ (N=8)**	***Mkp-5*$^{+/+}$ (N=6)**
**IVSd (mm)**	0.73 ± 0.02	0.75 ± 0.04
**IVSs (mm)**	1.15 ± 0.04	1.20 ± 0.04
**LVIDd (mm)**	4.16 ± 0.08	4.18 ± 0.15
**LVIDs (mm)**	2.94 ± 0.08	2.86 ± 0.17
**LVPWd (mm)**	0.76 ± 0.03	0.75 ± 0.04
**LVPWs (mm)**	1.10 ± 0.05	1.10 ± 0.04
**EF (%)**	56.90 ± 1.68	60.17 ± 3.18
**FS (%)**	29.59 ± 1.11	31.99 ± 2.10
**LV mass**	115.33 ± 6.26	117.53 ± 12.61
**LV mass corrected**	92.26 ± 5.01	94.03 ± 10.09
**LV Vol d (µl)**	77.30 ± 3.42	78.59 ± 7.04
**LV Vol s (µl)**	33.48 ± 2.40	32.07 ± 4.84

Data are mean ± s.e.m. IVSd: interventricular septum thickness in diastole; IVSs: interventricular septum thickness in systole; LVIDd: left ventricular end-diastolic diameter; LVIDs: left ventricular end-systolic diameter; LVPWd: left ventricle posterior wall thickness in diastole/systole; LVPWs: left ventricle posterior wall thickness in diastole/systole; EF: ejection fraction; FS: fractional shortening; LV Vol d: left ventricular end-diastolic volume; LV Vol s: left ventricular end-systolic volume.

Supplementary Table 3: qRT-PCR array analysis of fibrosis-related genes in FACS-sorted Ly6C^{low} macrophages *Mkp-5*$^{+/+}$ and *Mkp-5*$^{-/-}$ myocardium 1 week after TAC.

Gene name	Gene symbols	MKP5$^{-/-}$ *v.s.* WT fold change*
Matrix metallopeptidase 9	*Mmp-9*	8.90
Latent transforming growth factor beta binding protein 1	*Ltbp1*	4.53
Integrin beta 6	*Itgb6*	2.99
Interleukin 1 alpha	*Il1a*	2.79
Plasminogen activator, urokinase	*Plau*	2.31
Decorin	*Dcn*	1.91
Matrix metallopeptidase 13	*Mmp-13*	1.80
Matrix metallopeptidase 1a (interstitial collagenase)	*Mmp-1a*	1.77
Tumor necrosis factor	*Tnf*	1.75
Cellular communication network factor 2	*Ctgf*	1.63
Myelocytomatosis oncogene	*Myc*	1.59
Platelet derived growth factor, alpha	*Pdgfa*	1.58
Plasminogen activator, tissue	*Plat*	1.48
B cell leukemia/lymphoma 2	*Bcl2*	1.42
Interleukin 10	*Il10*	1.42
Epidermal growth factor	*Egf*	1.37
Tissue inhibitor of metalloproteinase 1	*Timp1*	1.36
Trans-acting transcription factor 1	*Sp1*	1.35
Chemokine (C-C motif) ligand 3	*Ccl3*	1.28
CCAAT/enhancer binding protein (C/EBP), beta	*Cebpb*	1.24
Thymoma viral proto-oncogene 1	*Akt1*	1.22
Endoglin	*Eng*	1.19
SMAD family member 7	*Smad7*	1.19
Collagen, type III, alpha 1	*Col3a1*	1.18
Integrin linked kinase	*Ilk*	1.16
Serine (or cysteine) peptidase inhibitor, clade E, member 1	*Serpine1*	1.16
Actin alpha 2, smooth muscle, aorta	*Acta2*	1.14
Nuclear factor of kappa light polypeptide gene enhancer in B cells 1, p105	*Nfkb1*	1.12
Transforming growth factor, beta receptor II	*Tgfbr2*	1.08
Chemokine (C-C motif) receptor 2	*Ccr2*	1.05
Transforming growth factor, beta receptor I	*Tgfbr1*	1.03
Matrix metallopeptidase 14 (membrane-inserted)	*Mmp14*	1.01
Lysyl oxidase	*Lox*	1.01
Interleukin 1 beta	*Il1b*	1.00
Integrin beta 1 (fibronectin receptor beta)	*Itgb1*	1.00
Integrin beta 5	*Itgb5*	0.99
TGFB-induced factor homeobox 1	*Tgif1*	0.97
SMAD family member 3	*Smad3*	0.95
Integrin alpha V	*Itgav*	0.93

Collagen, type I, alpha 2	*Col1a2*	0.92
SMAD family member 2	*Smad2*	0.89
Serine (or cysteine) peptidase inhibitor, clade H, member 1	*Serpinh1*	0.88
Bone morphogenetic protein 7	*Bmp7*	0.88
Endothelin 1	*Edn1*	0.86
Transforming growth factor, beta 3	*Tgfb3*	0.84
Platelet derived growth factor, B polypeptide	*Pdgfb*	0.80
Chemokine (C-X-C motif) receptor 4	*Cxcr4*	0.77
Transforming growth factor, beta 1	*Tgfb1*	0.76
Jun proto-oncogene	*Jun*	0.72
SMAD family member 4	*Smad4*	0.70
Tissue inhibitor of metalloproteinase 2	*Timp2*	0.70
Hepatocyte growth factor	*Hgf*	0.69
Tissue inhibitor of metalloproteinase 3	*Timp3*	0.67
Chemokine (C-C motif) ligand 12	*Ccl12*	0.64
Integrin alpha 1	*Itga1*	0.58
Integrin beta 3	*Itgb3*	0.57
Signal transducer and activator of transcription 1	*Stat1*	0.57
Caveolin 1, caveolae protein	*Cav1*	0.47
Matrix metallopeptidase 2	*MMP2*	0.45
Thrombospondin 1	*Thbs1*	0.37
Vascular endothelial growth factor A	*Vegfa*	0.35

*Fold change ($2^{-\Delta\Delta CT}$) was calculated by the normalized gene expression ($2^{-\Delta CT}$) in the *Mkp-5*$^{-/-}$ samples divided by the normalized gene expression ($2^{-\Delta CT}$) in the *Mkp-5*$^{+/+}$ control samples.

Supplementary Figure S1

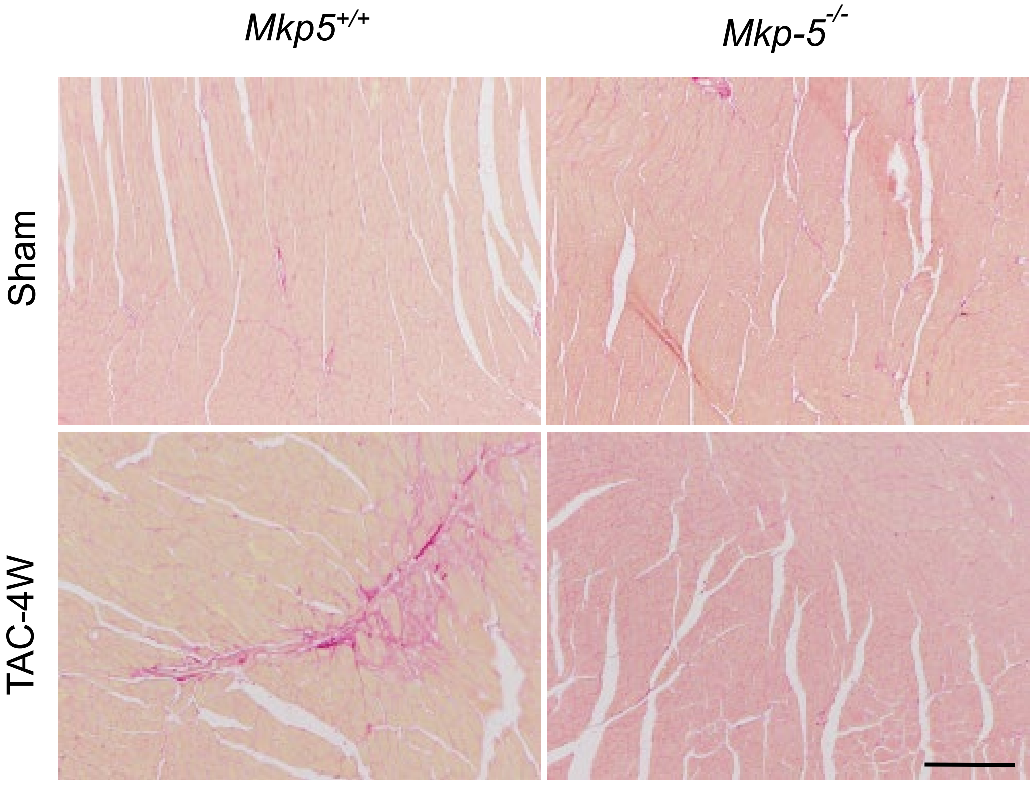

Figure S1. MKP-5 deficiency reduces cardiac fibrosis at 4 weeks after TAC. Representative images of Sirius Red-stained sections of *Mkp-5*$^{+/+}$ and *Mkp-5*$^{-/-}$ mouse hearts at 4 weeks after sham or TAC operation (scale bar, 100 μm).

Supplementary Figure S2

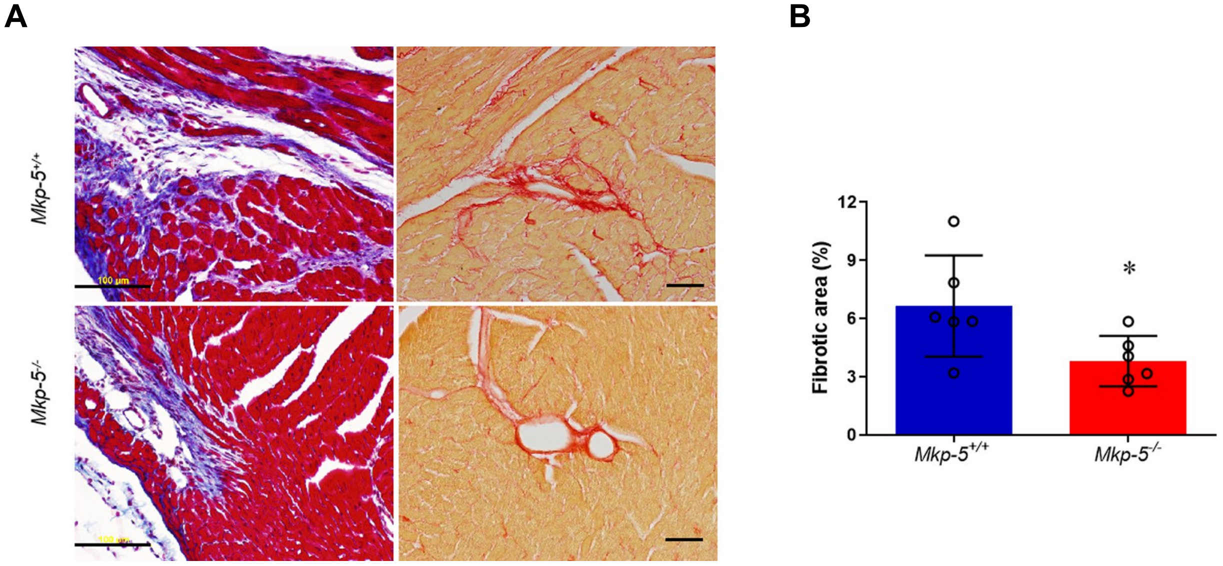

Figure S2. MKP-5 deficiency attenuates myocardial fibrosis after MI. (A) Representative Masson's trichrome- (left panel) and Sirius Red-stained (right panel) sections of *Mkp-5*$^{+/+}$ and *Mkp-5*$^{-/-}$ mouse hearts at 4 weeks after MI (scale bar 100 μm). **(B)** As indicated by Sirius Red staining, the total cardiac fibrotic area was quantified using sections of *Mkp-5*$^{+/+}$ and *Mkp-5*$^{-/-}$ mouse hearts at 4 weeks after MI (n=6 mice). *$P < 0.05$ vs *Mkp-5*$^{+/+}$. Data are mean ± SEM. Two-tailed, unpaired Student's *t*-test was used for statistical analysis.

Supplementary Figure S3

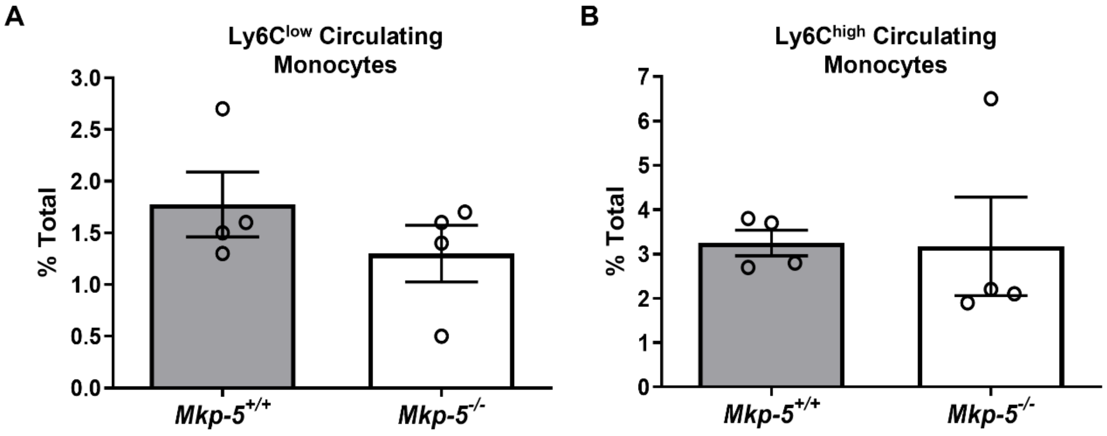

Figure S3. Effect of MKP-5 deficiency on circulating monocyte subsets after TAC. (A, B) Quantification of $Ly6C^{low}$ monocyte subset **(A)** and $Ly6C^{hi}$ monocyte subset **(B)** in the blood of *Mkp-5*$^{+/+}$ and *Mkp-5*$^{-/-}$ mice at 1 week after TAC (n=4). Data are mean ± SEM. Two-tailed, unpaired Student's *t*-test was used for statistical analysis.

Supplementary Figure S4

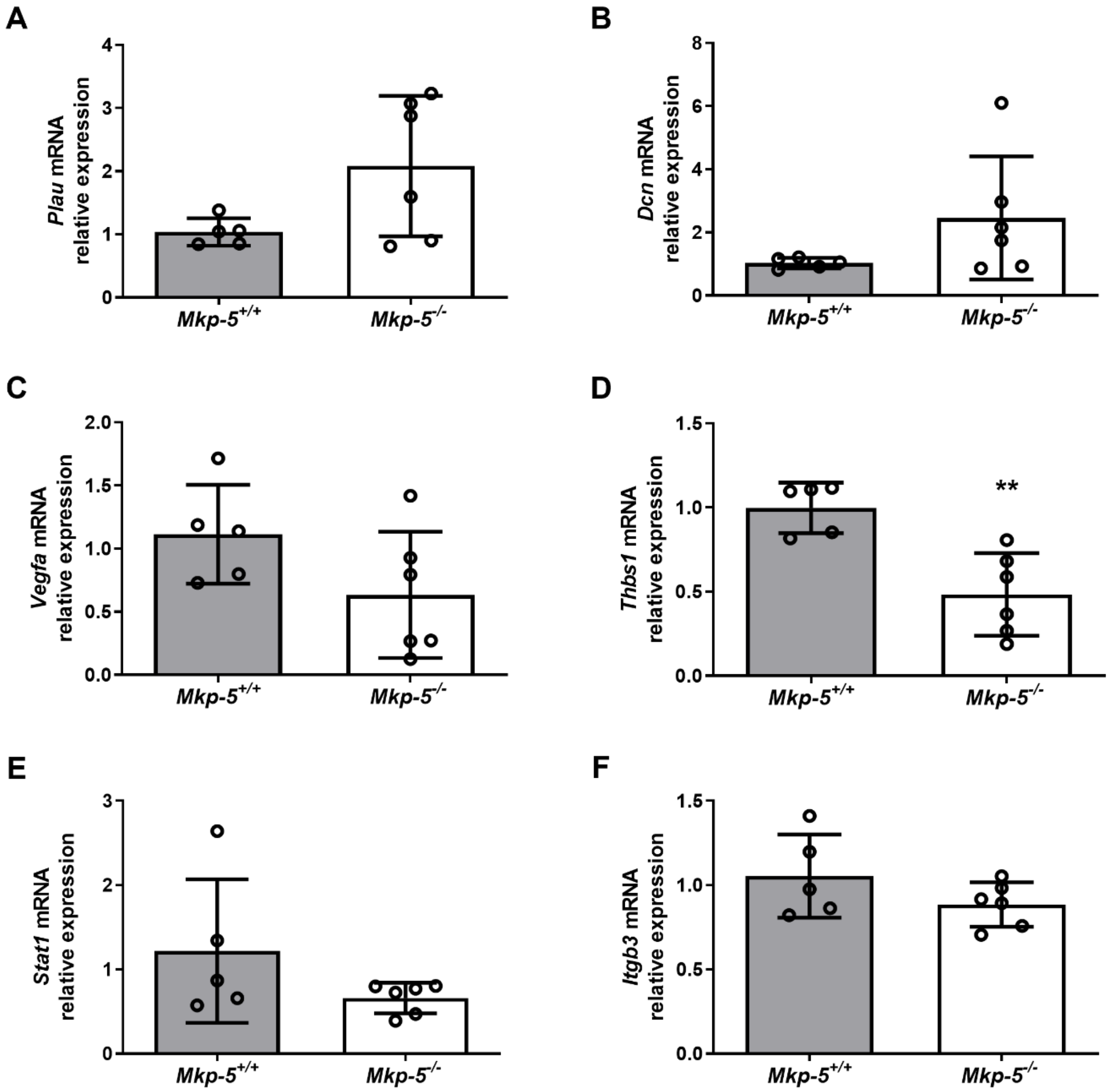

Figure S4. Validation of fibrosis qRT-PCR array results. (A-F) qRT-PCR analysis of *Plau* **(A)**, *Dcn* **(B)**, Vegfa **(C)**, *Thbs1* **(D)**, *Stat1* **(E)**, and Itgb3 **(F)** in Ly6C^{low} cardiac macrophages isolated from a second cohort of *Mkp-5*$^{+/+}$ and *Mkp-5*$^{-/-}$ mice that underwent 1 week of TAC (n=5-6). $^{**}P$ < 0.01 vs *Mkp-5*$^{+/+}$. Data are mean ± SEM. Two-tailed, unpaired Student's *t*-test was used for statistical analysis.
